# Effector Memory Th1 CD4 T Cells Are Maintained in a Mouse Model of Chronic Malaria

**DOI:** 10.1371/journal.ppat.1001208

**Published:** 2010-11-24

**Authors:** Robin Stephens, Jean Langhorne

**Affiliations:** Division of Parasitology, MRC National Institute for Medical Research, London, United Kingdom; McGill University, Canada

## Abstract

Protection against malaria often decays in the absence of infection, suggesting that protective immunological memory depends on stimulation. Here we have used CD4^+^ T cells from a transgenic mouse carrying a T cell receptor specific for a malaria protein, Merozoite Surface Protein-1, to investigate memory in a *Plasmodium chabaudi* infection. CD4^+^ memory T cells (CD44^hi^IL-7Rα^+^) developed during the chronic infection, and were readily distinguishable from effector (CD62L^lo^IL-7Rα^−^) cells in acute infection. On the basis of cell surface phenotype, we classified memory CD4^+^ T cells into three subsets: central memory, and early and late effector memory cells, and found that early effector memory cells (CD62L^lo^CD27^+^) dominated the chronic infection. We demonstrate a linear pathway of differentiation from central memory to early and then late effector memory cells. In adoptive transfer, CD44^hi^ memory cells from chronically infected mice were more effective at delaying and reducing parasitemia and pathology than memory cells from drug-treated mice without chronic infection, and contained a greater proportion of effector cells producing IFN-γ and TNFα, which may have contributed to the enhanced protection. These findings may explain the observation that in humans with chronic malaria, activated effector memory cells are best maintained in conditions of repeated exposure.

## Author Summary

Protective immunity against malaria develops only after several infections and can be lost on leaving an area in which malaria is transmitted. This suggests that the chronic infection may maintain the protective immune response. In this paper we have used a mouse model of a blood-stage malaria infection to examine the memory response of CD4^+^ T cells during chronic infection. These T cells are required for protective immunity, and also play a part in the inflammatory response that gives rise to malaria disease. Understanding what constitutes a protective CD4^+^ T cell may help us design more protective vaccines. We show that these memory CD4^+^ T cells persist in an activated state, produce the inflammatory cytokines TNFα and IFN-γ, and are more protective than “resting” memory CD4^+^ T cells obtained from mice in which the infection has been eliminated. This may explain why people are better protected against malaria disease when they are infected frequently.

## Introduction

Protective immunity to malaria develops only after repeated infections; although protection from homologous infection [1] and lethal malaria occurs after one to two infections [2]. Immunity to infection can persist for years; however, clinical immunity can be lost on emigration away from endemic areas, and high levels of exposure lead to lower disease prevalence than lower exposure [3]. Furthermore, human vaccine trials and mouse models have shown that immunity decays both with time after vaccination and that treatment of infection reduces protection [4], [5], [6]. These observations suggest that continuous exposure to the parasite may be required for the maintenance of immunological protection from malaria, as has also been suggested in *Leishmania* and other chronic infections [7], [8]. Recent work with *Plasmodium chabaudi* demonstrated that the decay of protection is replicated in mouse models and that this may be determined by a decay in memory T cell (Tmem) function [5].

Adaptive immunity to infection develops by accrual of antigen-experienced memory cells. In the absence of chronic infection, resting, antigen-independent memory T cells reside in secondary lymphoid organs; however, in chronic infection, memory cells and effector cells may be continually generated [9], and may even expand the memory cell pool [10]. Central memory T cells (Tcm, [11]), defined by high levels of expression of CD62L, have been shown to be protective in various infections [12], [13]. However, infection with *Plasmodium* liver-stages and other chronic infections have been shown to primarily produce effector memory and effector CD8^+^ T cells [14], [15], which are also protective [12], [16]. In humans, CD8+ effector memory (Tem) cells have been subdivided with activation markers into early and late subsets, with different subsets predominating in different infections, however, it has not yet been determined how they are derived [17], [18], [19]. In some chronic infections where high pathogen loads persist, such as HIV and LCMV, chronic stimulation leads to functional impairment or exhaustion of CD8^+^ T cells, and production of IL-10, which slows clearance of the pathogen [20], while in other infections, such as HCV, virus-specific CD8^+^ memory T cells actually accumulate [9], [21].

While there have been relatively few studies of CD4^+^ T cell memory in malaria, it is known that immunity to the blood stages of *Plasmodium* is dependent on both CD4^+^ T cells and B cells [22], and the presence of *Plasmodium*-specific CD4+ T cells, in some cases, has been shown to correlate with clinical immunity [23]. However, it has been shown that *P. yoelii* and *P. berghei* infections can lead to deletion of specific CD4^+^ T cells generated by vaccination [24], and a recent study showed that protective CD4^+^ T cell memory decays after 6.5 months in *P. chabaudi* infection [5], suggesting some impairment of long-lived immunological memory in blood-stage malaria infections. Therefore it is critical to understand more about the generation and maintenance of memory T cells in malaria to improve our capacity to generate long-lived protection by vaccination.

Here we have investigated the development of *Plasmodium*-specific CD4^+^ T cells using a transgenic mouse with CD4^+^ T cells specific for a peptide within Merozoite Surface Protein-1 (MSP1, [25]), the B5 T cell receptor Transgenic (B5 TCR Tg). MSP1 is expressed on the surface of merozoites and is a candidate antigen for inclusion in a blood-stage malaria vaccine. We establish that a heterogeneous memory CD4^+^ T cell population develops after a primary infection, and is composed predominantly of early effector memory T cells (TemE). We demonstrate a pathway of differentiation from central memory to early and then late effector memory CD4 T cells by adoptively transferring these three subsets into *P. chabaudi*-infected RAG° mice. We also show that upon adoptive transfer, memory CD4^+^ T cells from chronically infected mice delay the onset of parasitemia and protect better against pathology than rested memory cells, suggesting that continuous stimulation enhances the effector function and protective capacity of memory T cells in malaria, correlating with their enhanced Th1 cytokine production. While we have previously shown the importance of B cells and antibodies in clearance of primary infection, the current studies suggest a role for cytokine production by memory T cells in long-term protection. This may be one of the reasons why people are better protected against malaria when they are re-infected frequently.

## Results

In order to identify malaria-specific CD4^+^ T memory cells during malaria, we have followed the response of *P. chabaudi*-specific transgenic CD4^+^ T cells, which carry a TCR recognising a peptide of Merozoite Surface Protein-1 (B5 Tg, [25]), throughout a blood-stage infection of *P. chabaudi* in mice. This infection is characterised by an acute phase with a peak parasitemia 8–10 days post-infection of approximately 25% infected erythrocytes. After the acute phase, parasitemia is reduced but not eliminated and can persist as a low-grade chronic infection for up to 3 months [6]. Purified naïve (CD44^lo^CD25^−^), MSP1-specific B5 TCR Tg CD4^+^ T cells (Thy1.2^+^) were labelled with CFSE and injected into BALB/c Thy1.1 mice (2×10^6^ per mouse). The recipient mice were infected with *P. chabaudi* the following day, and the transferred B5 Tg T cells were followed by flow cytometry for 60 days after infection.

**Figure 1:**
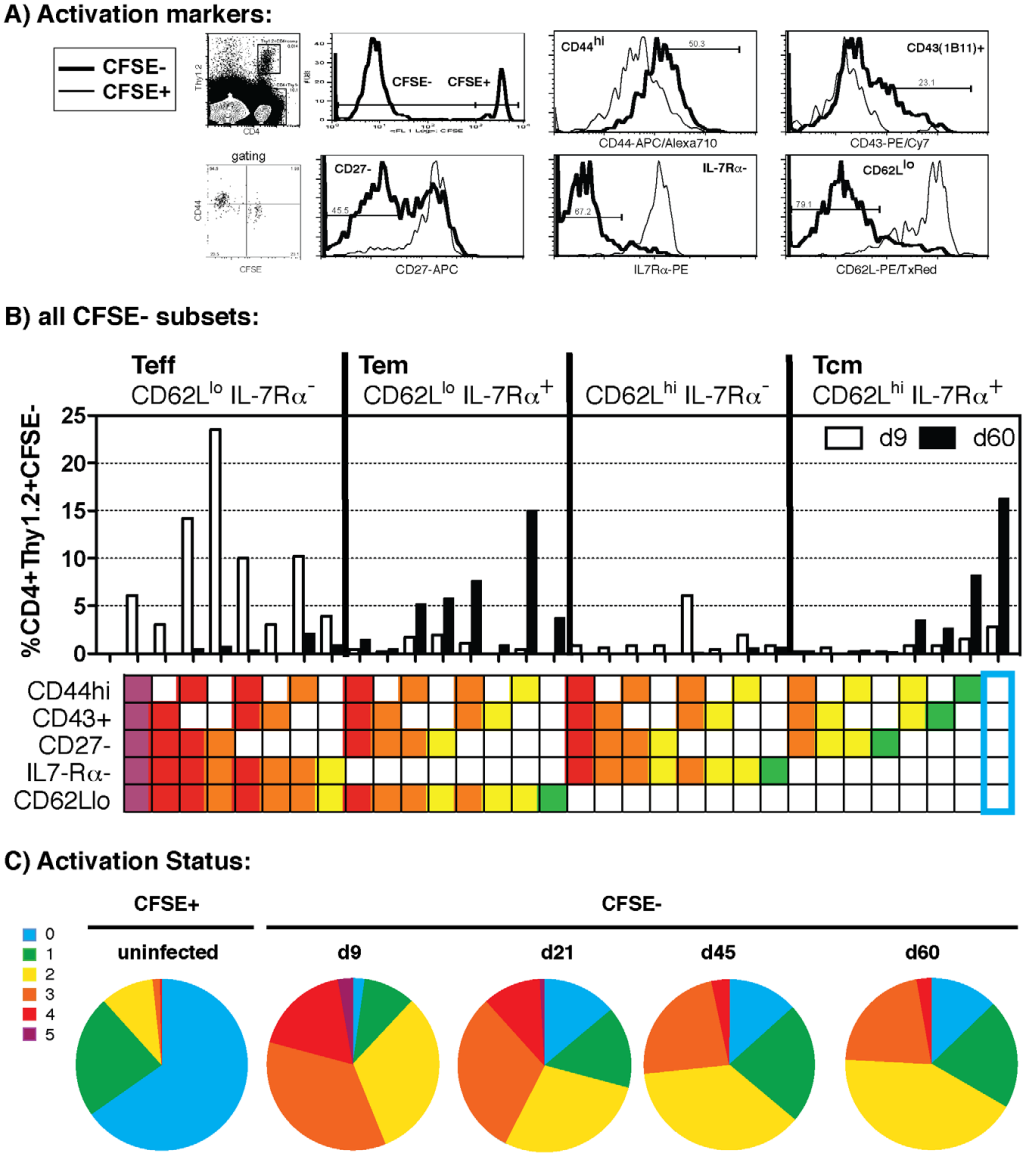
Memory T cells with an activated phenotype can be distinguished from effector T cells. Sorted Naïve (CD44^lo^CD25^−^), CFSE-labelled B5 T cells (2×10^6^) were seeded into congenic Thy1.1^+^ mice. Mice were infected with 10^5^ *P. chabaudi* iRBC, sacrificed and splenocytes stained with anti-CD4, Thy1.2 and activation markers CD44, CD43, CD27, IL7Rα and CD62L at different times post infection. **A**) Divided B5 T cells (CFSE^neg^ Thy1.2^+^, left plot and histogram) were analyzed for expression of the activation markers on day 9 post-infection by multiparameter flow cytometry. Histograms show gating for the activation markers. CFSE^neg^ B5 Tg effector T cells (Teff) are CD44^int-hi^, IL-7Rα^−^, CD62L^lo^, and can be CD43^+/−^, and CD27^+/−^. **B**) CFSE^neg^ (divided) B5 Tg T cells were boolean gated into 32 possible subsets based on their expression all five markers. The percentage of cells expressing each combination of markers for all of the subsets at day 9 (white bars) and day 60 (black bars) are shown in the histograms. Boxes below indicate the marker listed to the left. The blue box on the right indicates the group with none of the markers of activation. **C**) CFSE^neg^ Tg cells were pooled into groups according to the number of activation markers expressed (color coded for 0–5 markers of activation as shown in the panel on the left, with colors ranging from blue: no activation markers; to purple, 5 activation markers), and the percentage of cells in each group is shown in a pie chart to indicate the relative activation state on a given day post-infection. A representative experiment of 3 performed is shown. The values shown are for 2–5 mice at each time point, cytometry data were concatenated. Experiment was repeated twice with similar results.

The acute *P. chabaudi* infection lasting about 20 days, and the subsequent low-level chronic infection may result in the continuous generation of effector cells, as has been reported in other persistent infections [9], [21], [26]. Therefore, we utilized multiparameter flow cytometry and the activation and memory markers, CD44, CD43, CD27, IL-7Rα and CD62L [16], [27], [28] to distinguish between CD4^+^ effector and memory subsets. At day 9 of infection, the majority of Tg cells had divided (CFSE^neg^, FACS gating showing level of CD44 expression correlating with cell division is shown in **Figure S1**), and were defined as effector cells (Teff) CD62L^lo^, IL-7Rα^−^, CD43^+^, and CD27^−^, and CD44^int^ (**Figure 1A**). Flow cytometry data gated on CD4^+^Thy1.2^+^ CFSE^neg^ divided B5 Tg cells were collected at several time-points during the infection, and subjected to boolean gating analysis, which distributed them into the 32 possible subsets generated by testing every possible combination of the 5 activation markers. This is shown in the histogram in **Figure 1B**, where the percentage of the divided B5 Tg CD4^+^ T cells falling into each subset is illustrated. Strikingly, this unbiased analysis identified effector cells as CD62L^lo^IL-7Rα^−^ during the acute parasitemia at day 9, while memory cells were easily distinguished by their upregulation of IL-7Rα, as seen in the majority of divided, CFSE^neg^ B5 Tg cells by day 60, which fell into both the effector (Tem) and central (Tcm) memory categories as indicated by their expression of CD62L. Various subsets could be seen within these major populations, and the other three markers, CD44, CD43, CD27, were used to determine the overall activation status of the cells. By grouping subsets based on the number of activation markers they express, we constructed pie charts to demonstrate graphically how activated the MSP1-specific cells were throughout the *P. chabaudi* infection (**Figure 1C**). More than 80% of the Tg T cells expressed markers of activation at day 9, and interestingly, many malaria-specific cells remained activated even at day 21, when the parasite was cleared to below 0.01% of erythrocytes infected. At day 45, the activation status of the cells appeared to have stabilized, so that even at day 60, 25% of the cells maintained three or more markers of activation. This suggests that a large proportion of the MSP1-specific Tg T cells that persisted late into the chronic phase of infection remained considerably activated and did not become resting or central memory cells in this time.

In these studies, when 2×10^6^ B5 Tg CD4+ T cells were transferred into the recipient mice, more than 70% of the specific cells divided, as shown in **Figure 1A**. We focused on divided cells (CFSE^neg^) as they represent the specific response, however, it is possible that transfer of this number of cells may have led to non-physiological responses [29]. We therefore transferred fewer T cells (**Figure S2A**) and were able to detect them by enriching Thy1.2^+^ cells before analysis and utilizing the enhanced signal to noise ratio of double staining to detect the small numbers of cells. We could see expansion of 30,000 or 5,000 naïve purified T cells at the peak of infection (day 6) in the spleen, with some cells still remaining undivided (**Figure S2B**), as in the transfer of 2×10^6^ cells. By day 60 more cells divided when fewer cells were transferred, as previously reported, and there was a similar distribution of memory T cell subsets as with more cells. When less than 10^6^ cells were transferred, less than 50 B5 cells were collected at day 60 necessitating concatenation of the FACS data for visualization. Transferring more cells allowed us to analyze the data from each mouse separately, concatenating only for display. Therefore it was not possible to carry out further experiments with lower numbers of cells and attain statistically meaningful data. When few cells are transferred no transferred cells are detectable in uninfected mice after two months, consistent with the data of others [29], and suggesting that the transgenic cells are not expanding in a malaria-antigen-independent manner. The approach of transferring 2×10^6^ cells was further validated by the consistency of data from 5 individual mice in **Figure S3**. Importantly, transfer of 2×10^6^ cells resulted in a physiological response, in that the small fraction that survives (0.01%) was the same proportion as MSP-1 responsive T cells in wild-type mice, as assessed previously by limiting dilution analysis (0.011% (1/8800) of CD4^+^ T cells from BALB/c mice respond to B5-containing fragment of MSP-1 after ten weeks of *P. chabaudi* infection [30]).

### Effector CD4^+^ T cells persist in *P. chabaudi* infection and effector memory cells predominate

**Figure 2:**
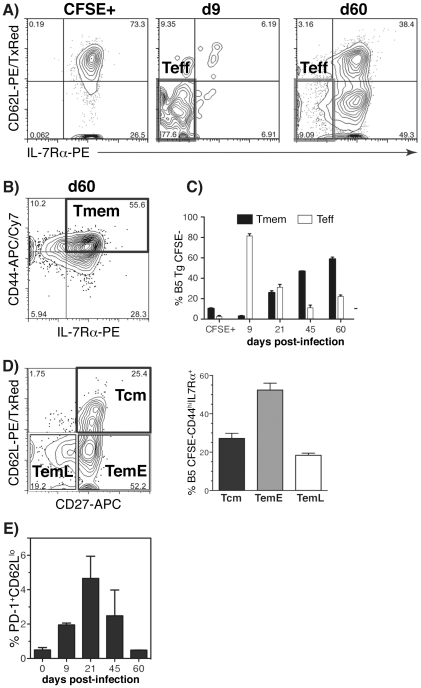
Effector CD4^+^ T cells persist in *P. chabaudi* infection and early effector memory cells predominate. Naïve (CD44^lo^CD25^−^), CFSE-labelled B5 T cells (2×10^6^) were seeded into congenic Thy1.1^+^ mice, which were then infected with 10^5^ *P. chabaudi* iRBC. **A**) Divided B5 cells (CFSE^neg^) were analyzed for expression of CD62L and IL-7Rα to measure the percentages of **Teff** (CD62L^lo^, IL-7Rα^−^) in the spleen on day 9 (middle panel) and day 60 (right panel). As an internal negative control, naïve cells (CFSE^+^) from uninfected mice are shown (left panel). **B**) CD44^hi^IL-7Rα^+^ memory cells are shown in the divided CFSE^neg^ population. **C**) Kinetics of appearance of Teff and Tmem as defined in A) and B) by percentage (left panel) or number (×10^3^, right panel). **D**) Tmem were subdivided using CD62L and CD27 to measure central (**Tcm**, CD62L^hi^CD27^+^), and early effector memory cells (**TemE**, CD62L^lo^CD27^+^) as well as CD27^−^ late effector memory T cells (**TemL**, CD62L^lo^CD27^−^) shown here at day 60 of infection. The relative proportions of Tcm, TemE and TemL of infection are shown in the right graph. **E**) PD-1 expression on B5 Transgenic CD4^+^ T cells seeded in Thy1.1 congenic mice at different times during *P. chabaudi* infection. All PD-1^+^ cells were CD62L^lo^. Graphs represent the means and SEM of 2 to 5 mice per time point. Plots show CD4^+^Thy1.2^+^CFSE^neg^ cells concatenated from 2–5 mice per time point (gated as shown in Figure 1A, raw data for B, D shown in Figure S3). Experiments were repeated three times with similar results.

Effector and memory cells amongst the transferred Tg CD4 T cells were further analyzed using the expression profile, IL-7Rα^−^CD62L^lo^, described above for effector cells (**Figure 2A**) and CD44^hi^IL-7Rα^+^ (**Figure 2B**, left contour plot) for memory T cells. As shown graphically in **Figure 2C**, at day 9 of infection, these effector CD4^+^ T cells accounted for approximately 75–80% of the MSP1-specific Tg T cell population, were still a substantial proportion of the total B5 T cell population at day 21, and despite low parasitemias, some even persist to day 60. CD44^hi^IL-7Rα^+^ CD4 memory cells were present by day 21 when parasitemia is very low, but memory cells did not become the dominant population until day 45. This is important as it suggests that the memory phase where memory T cells dominate over effector cells may be delayed by chronic low-level infection.

Human CD8^+^ effector memory T cells observed in various virus infections have been subdivided further into “early”, “intermediate” and “late” based on differential expression of CD28 and CD27 [18]. We wanted to determine whether the CD44^hi^IL-7Rα^+^ Tg CD4+ memory cells in *P. chabaudi* infection also contained subpopulations suggesting differential stimulation, or stages of differentiation. CD28 could not be used in our experiments as a marker for further subdivision of Tem subsets, as expression of this molecule is not up-regulated on mouse T cells with activation [17], [18]. We therefore defined subsets within the IL-7Rα^+^ memory Tg cells using CD27 and CD62L. CFSE^neg^ (divided) B5 T cells were gated on CD44^hi^IL-7Rα^+^ memory cells and expression of CD27, and CD62L analyzed on these cells to show the distribution of central and effector memory T cells (**Figure 2D**, left contour plot). There were two effector memory subsets, which correlated with early and late effector memory (TemE, TemL). This analysis on day 60 post-infection showed that TemE B5 Tg cells (CD27^+^, CD62L^−^) dominated the response (**Figure 2D**, graph). Both the proportion of CD44^hi^ IL-7Rα^+^ memory T cells and the subsets defined by CD27 and CD62L remained similar on days 45 and 60 (data not shown). We also observed the development and trafficking of memory T cells in the lymph nodes and saw both central and effector memory cells, while liver contained only effector memory cells (**Figure S4**). Memory cells were also observed in the bone marrow in small numbers (data not shown).

In many, but not all, persisting infections, T cells have been shown to express inhibitory receptors such as PD-1 (CD279) and KLRG1. These correlate with dysfunction of the T cells called exhaustion, and can regulate pathogenic responses [31], [32]. However, although *P. chabaudi* infection in mice has a prolonged acute parasitemia and a long chronic phase, few PD-1^+^ effector (**Figure 2E**) Tg CD4^+^ T cells (all CD62L^lo^, maximum of 5.7% at day 21) were transiently present, and no KLRG-1+ cells were observed (data not shown). This suggests that terminal differentiation of effector cells is taking place normally, and that exhaustion of CD4^+^ T cells is not a problem in this malaria infection, but the data show the memory cells remain in an activated state. Therefore, we investigated the pathway of differentiation that leads to this state.

### Differentiation of malaria-specific CD4^+^ T cell memory subsets

**Figure 3:**
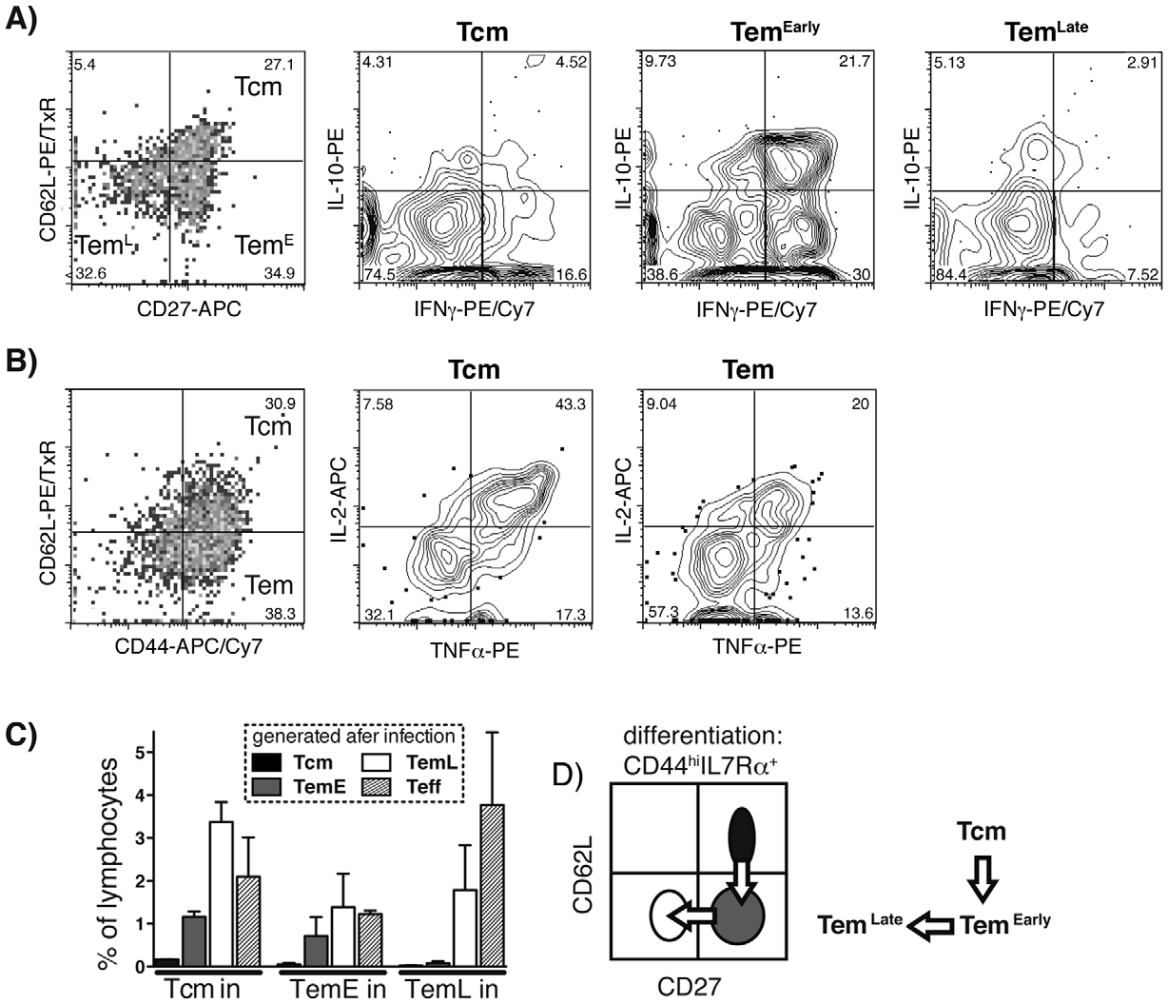
Heterogeneity of cytokine production and differentiation potential of the memory CD4^+^ T-cell subsets in chronic *P. chabaudi* infection. **A**) and **B**) Naïve (CD44^lo^CD25^−^), CFSE-labelled B5 T cells (2×10^6^) were seeded into congenic Thy1.1^+^ mice to follow the antigen-specific cytokine response in memory cell subsets. Mice were infected with 10^5^ *P. chabaudi* infected RBC and CD4^+^ T cells analyzed 60 days later. Divided B5 cells (CD44^hi^ CFSE^neg^) were analyzed for **A**) expression of CD27 and CD62L to identify memory T cell subsets (**Tcm**, CD62L^hi^CD27^+^), (Tem^early^ (**TemE**), CD62L^lo^CD27^+^) and (Tem^late^ (**TemL**), CD62L^lo^CD27^−^) and production of IL-10 and IFN-γ in each subset. **B**) Divided B5 T cells (CFSE^neg^) were analyzed for CD44 and CD62L to identify **Tem** (CD44^hi^CD62L^lo^) and **Tcm** (CD44^hi^CD62L^hi^) and production of IL-2 and TNF-α. These data are representative of 5 individual mice. **C**) and **D**) Purified memory T cell subsets (CD4^+^CD44^hi^IL-7Rα^+^); **Tcm**, (CD62L^hi^CD27^+^), (**TemE**, CD62L^lo^CD27^+^) and (**TemL**, CD62L^lo^CD27^+^) were transferred (10^5^) into RAG° mice which were then infected with *P. chabaudi*. Spleens were analysed on day 38 after infection by multi-parameter FACS analysis; **C**) memory cell composition on recovery, Tcm, TemE and TemL, as defined in **A**), of CD4^+^ T cells, shown as percentage of lymphocytes; **D**) Proposed linear differentiation pathway for memory T cell subsets based on the data in **C**).

As early (CD27^+^ CD62L^−^) and late (CD27^−^ CD62L^−^) effector memory cell subsets (TemE and TemL) have not previously been studied in mouse CD4^+^ T cell populations, we investigated the cytokine profiles and differentiation potential of each of the three subsets in this *P. chabaudi* infection. Using TAPI-2, an inhibitor of the metalloproteases that cleave CD62L and TNFα [33], we were able to perform intracellular cytokine staining on memory T cells at day 60 post-infection whilst maintaining expression of CD62L. MSP1-specific Tcm, TemE and TemL cells were identified using CD44, CD62L and CD27, as described above, and their IFN-γ and IL-10 profiles analyzed (**Figure 3A**). Interestingly, while Tcm and TemL subsets contained small populations of IFN-γ^+^ cells and few IL-10^+^ cells; 52% (+/−4.1 SEM) of TemE cells made either IL-10 or IFN-γ, and 14% (+/−2.9 SEM) made both cytokines. Despite producing little IFN-γ or IL-10, almost 70% CD44^hi^CD62L^hi^ Tcm cells were capable of making TNF or IL-2. Tem on the other hand, contained fewer TNF and IL-2 positive cells (**Figure 3B**). It was not technically possible to analyse three cytokines together and still have sufficient available parameters to subdivide the Tem cell subsets further, or to include IL-7Rα to distinguish effector memory cells from effector cells (<10% on day 60).

In order to determine how the three memory T cell subsets relate to each other and to effector cells, and how they differentiate and survive in the face of constant exposure to infection, transfer experiments with the CD44^hi^IL-7Rα^+^ memory cell subsets were undertaken. We could not magnetically sort Thy1.2+ Tg cells from the spleen at day 60 due to hemozoin, an insoluble iron-containing catabolite of hemoglobin that makes phagocytes magnetic. Therefore, to analyze memory T cell differentiation and protection *in vivo*, we generated memory cells to test by infecting the B5 transgenic mouse. Interestingly, although 80% of the cells in this mouse are malaria-specific [25], a normal percentage of effector cells and memory cells was generated (**Figure S5**). Therefore, purified Tcm (CD62L^hi^), TemE (CD62L^lo^CD27^+^), or TemL (CD62L^lo^CD27^−^) from B5 TCR Tg mice (**Figure S6** shows the FACS profiles of the sorted cells), were transferred into RAG° mice, that were then infected with *P. chabaudi*. These experiments have not yet been done in the absence of infection to ascertain the effect of homeostatic proliferation in the RAG° recipients on the differentiation of memory T cells, however, as antigen-induced proliferation is faster, it is likely that it is the dominant pathway in chronic infection. The results showed that not many Tcm cells were present on day 38. Although some did survive and maintain their phenotype, they had largely differentiated into effector memory and effector cells (**Figure 3C**, Tcm); suggesting that Tcm have the intrinsic capacity to become TemE, TemL and Teff when stimulated to divide. By contrast, TemE had the capacity to expand and generate TemL and Teff cells but not Tcm; while TemL only generated more TemL and Teff cells. These data indicate that there is a linear differentiation pathway from central to early effector to late effector memory, in high-level chronic *P.chabaudi* infection (represented in **Figure 3D**), and that all subsets can generate Teff.

### Removal of the chronic *P. chabaudi* infection by chloroquine treatment reduces protective capacity of memory T cells

We have shown previously that chronically infected C57Bl/6 mice are more resistant to a second infection with the homologous strain of *P. chabaudi* than mice whose chronic infection had been eliminated with the anti-malarial drug chloroquine (CQ) [6]. This was also the case for *P. chabaudi* infections in BALB/c mice (**Figure S7A**). This suggests that memory B and T cells are affected by chronic infection in such a way that they are more effective when continually exposed to antigen.

To determine whether chronically stimulated memory T cells contribute to this enhanced protection, we assessed the ability of MSP1-specific CD4^+^ T cells to confer protection in adoptive transfer experiments into RAG° mice. As we found that the individual memory T cell subsets in chronically infected mice did not show differential protection, unfractionated Tg memory CD4^+^ T cells (CD4^+^CD44^hi^) from chronically infected or from previously infected and chloroquine-treated B5 transgenic mice, were transferred into RAG° mice together with immune B cells, as described previously [25], [34].

We first verified that chloroquine treatment had resulted in loss of the B5 antigen from the system, as well as eliminating the chronic infection as described [6] (**Figure S7B**). CFSE-labelled MSP-1-specific Tg CD4 T cells were injected into chronically infected or infected and drug-cured mice at day 45 and 60 post-infection, and recovered and analyzed after 3 days. Cell division of the Tg cells, measured by CFSE levels, indicated whether the MSP1 peptide was still being presented. Indeed, in the chronically infected B5 mice, the B5 MSP1 peptide was still presented on antigen-presenting cells at 45 and 60 days of infection as detected by proliferation of CFSE-labelled MSP1-specific B5 Tg T cells. By contrast, much less proliferation was observed in the Tg T cells recovered from drug-cured mice (**Figure S7B**), suggesting that after removal of parasites there was insufficient residual MSP1 antigen to stimulate Tg T cells and that we could consider these memory cells as “resting”.

**Figure 4:**
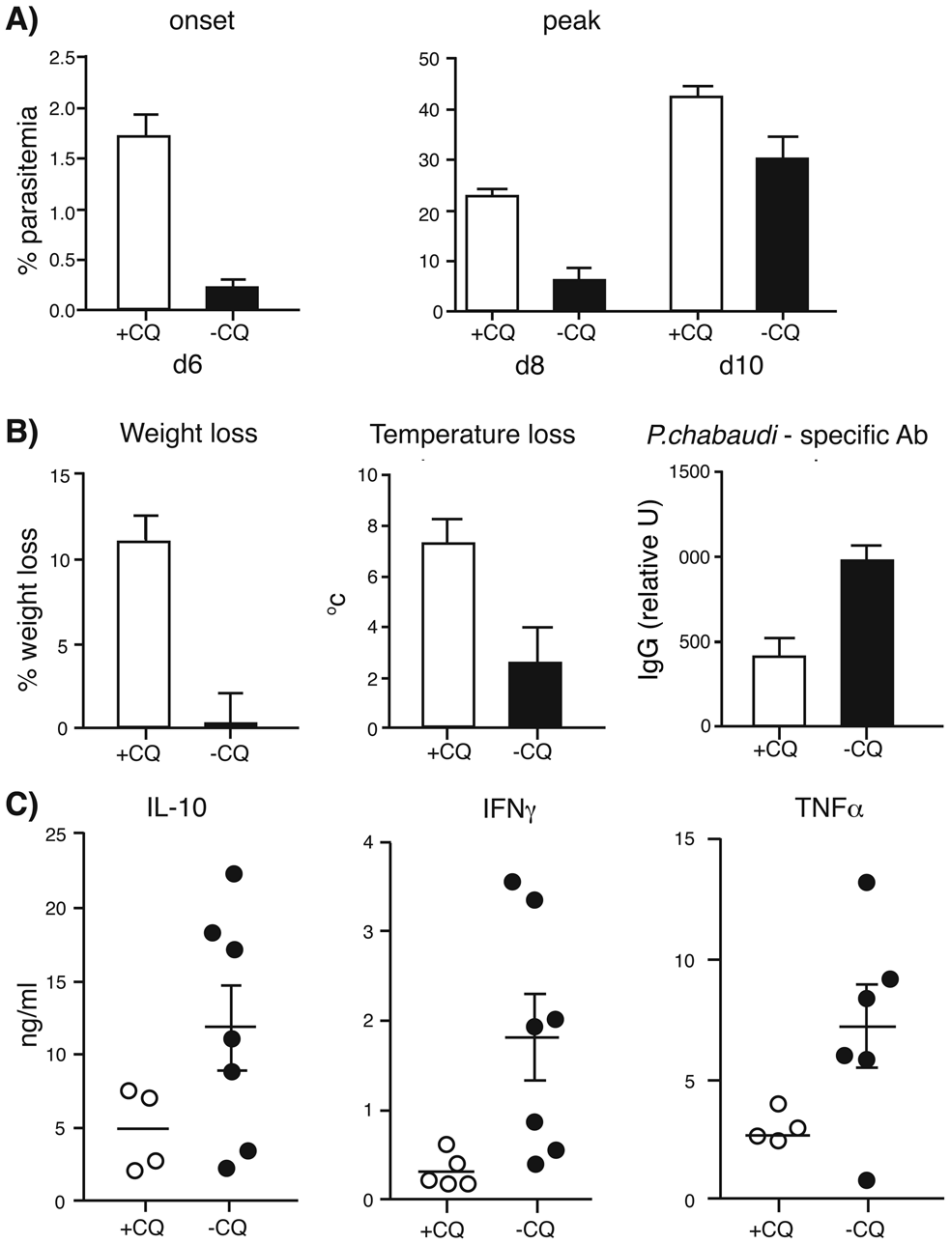
Chronically stimulated memory T cells protect immunodeficient mice from parasitemia and pathology better than rested memory T cells. MSP1-specific CD4^+^ B5 memory (CD44^hi^) or naïve (CD44^low^CD25^−^) T cells were purified from B5 TCR Tg mice infected 2.5 months previously and harbouring a chronic infection (**−CQ**, closed symbol/bar), or drug treated from days 30–34 to remove the chronic infection (**+CQ**, open symbol/bar). The cells (10^5^) were transferred into RAG° recipients together with immune B cells (10^7^) and the recipients infected with 5×10^4^ *P. chabaudi* iRBC. **A**) Parasitemia (geometric mean +/−SEM) is at onset of patent parasitemia (day 6, left graph), and at peak parasitemia (days 8 and 10, middle graph) **B**) Peak weight change is shown as a measure of cachexia (left graph), maximum loss of body temperature (middle graph) and *P.chabaudi* -specific IgG antibodies (day 21) measured in the serum of the transferred RAG° animals by ELISA. **C**) Serum IL-10, IFN-γ, and TNFα were measured on day 7 in RAG° mice receiving Tg cells from chronically infected (closed symbols) or drug-treated mice (open symbols). The data shown are the means and SEM of 4–7 mice.

After adoptive transfer of chronically simulated or rested CD4 T cells into recipient mice, there were two clear major measurable effects of these cell transfers on parasitemia (**Figure 4A**); a delay in appearance of early parasitemia, and a reduction in peak parasitemia. Mice receiving chronically stimulated memory T cells (−CQ) showed a delayed onset of parasitemia (**Figure 4A**, left graph), showing that these cells were more effective in controlling early parasite growth. Consistent with this, chronically stimulated memory T cells also reduced peak parasitemia (p = 0.02) compared with resting cells, (**Figure 4A**, right graph) or naïve cells (data not shown). Consistent with reduced parasitemia, mice receiving chronically stimulated cells exhibited less pathology than mice receiving rested memory T cells (**Figure 4B**) and naïve cells (not shown). Thus chronically stimulated malaria-specific memory T cells show an enhanced early protective effect with reduced parasitemias and reduced pathology compared with rested memory T cells. Furthermore, chronically stimulated B5 memory T cells provided more effective help for a *P. chabaudi*-specific antibody response than resting memory B5 cells (**Figure 4B**). RAG° mice receiving chronically stimulated T cells also had higher levels of TNFα, IFN-γ and IL-10, in plasma at day 7 of infection (**Figure 4C**), than those receiving rested T cells. These inflammatory cytokines may enhance parasite killing, while IL-10, may regulate their pathogenic effects, as has been described previously [35].

### Chronically stimulated memory T cells produce more Th1 cytokines than rested memory cells

As we have shown that the more protective chronically stimulated memory T cells resulted in larger amounts of circulating cytokines upon adoptive transfer into RAG° mice than rested memory T cells, we investigated whether this was also the case in the chronic infection itself, which might explain the greater protective efficacy of the chronically stimulated T cells. Memory cells making multiple cytokines have been proposed to correlate with the protectiveness of vaccines and the effectiveness of memory cells in various chronic infections that require T cells for clearance of the pathogen [36].

**Figure 5:**
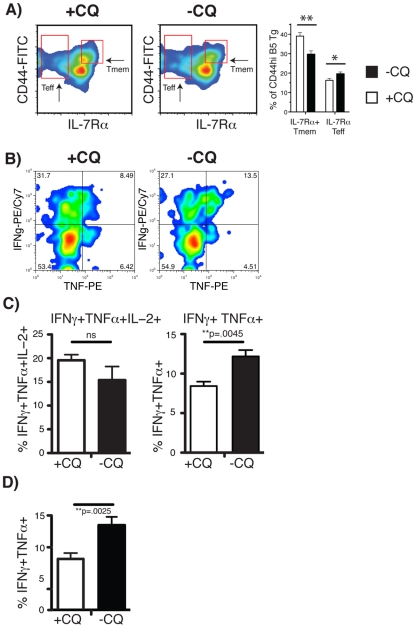
Treatment of a chronic *P. chabaudi* infection changes the phenotype of memory cells and their cytokine profiles towards Th1 late effector profile. CD4^+^ MSP1-specific B5 Tg T cells were seeded into Thy1.1 congenic mice to follow the antigen-specific response. Mice were infected with 10^5^ *Plasmodium chabaudi* and treated with Chloroquine (CQ) during days 30–34 to clear the chronic infection. Splenocytes were analyzed by flow cytometry on day 60 post-infection, and the FACS plots were gated on Thy1.2^+^CD4^+^ cells. **A**) Differences in the proportions of **Tmem**, (CD44^hi^ IL-7Rα^+^), and effector (**Teff**, CD44^hi^ IL-7Rα^−^) cells at day 60 between mice treated and not treated with chloroquine (**+CQ** and **−CQ** respectively). The percentage of IL-7Rα^+^ Tmem or IL-7Rα^−^ Teff within the CD44^hi^ cell population is shown as a graph (right). These differences were observed in 4 independent experiments. **B**) IFN-γ and TNFα production in Thy1.2^+^CD4+ CD44^hi^CD62L^lo^ B5 cells from chloroquine-treated and -untreated mice at day 60 post infection. **C**) Multiple cytokine producing B5 CD4 T cells from chloroquine-treated and untreated mice at day 60 post-infection. Percent double-producing TNFα^+^IFN-γ^+^IL-2^−^ and triple-producing IFN-γ^+^TNFα^+^IL-2^+^ are shown. **D**) IFNγ^+^ TNFα^+^ (IL-2^−^) double producing cells within the **Tem** subset of B5 at 60 days post-infection. The graphs show means and SEM from 5 mice. ** p≤.01 and * p≤.05 by student's t-test.

In line with the more rapid effect on parasitemia after adoptive transfer, there was a larger population of IL-7Rα^−^ effector T cells (Teff) in the chronically infected mice (**Figure 5A**, p = 0.038) compared with CQ-treated mice. Conversely, there was a significantly greater proportion of IL-7Rα^+^CD44^hi^ memory T cells in the B5 CD4^+^ T cell population from CQ-treated mice (+CQ, **Figure 5A**, p = 0.0073). The increase of effector cells in chronically infected mice and the decrease in memory T cells was also seen in the endogenous CD4^+^ population (**Figure S8**). We could not detect any reproducible differences in the activation markers of effector cells (as defined in **Figure 1**) between chronically stimulated and “rested” memory Tg CD4 T cells, nor in the composition of the memory cell subsets (as defined in **Figure 2C**).

To determine whether the proportion of memory T cells making multiple cytokines in *P. chabaudi* was different in chronically stimulated and “rested” Tg CD4 T cells, we compared their intracellular cytokine profiles on day 60 post-infection, as described above for **Figure 3**. Chronically stimulated (−CQ) Tg CD4 T cells at day 60 contained a higher proportion of cells producing both TNF and IFN-γ but not IL-2 than memory cells from drug-treated mice (**Figure 5B**, **Figure 5C**). The proportions of triple cytokine producing cells however were not significantly different (**Figure 5C**, left). The difference in the double-producing cells was mainly within the CD62L^lo^CD44^hi^ effector or effector memory cell population (**Figure 5D**). Our data suggest that the greater capacity of CD4^+^ T cells in chronic infection to control early parasitemia is due to the maintenance of Th1 effector cytokine potential by the effector or effector memory cells.

## Discussion

There is a real need to understand protective immune mechanisms in malaria in order to improve vaccination. Development of immunological memory to malaria infection develops over several exposures, and protection is short-lived in the absence of exposure to the parasite [37]. CD4^+^ T cell responses and specifically CD4^+^ T cell memory, which are of central importance in immunity to blood-stage *Plasmodium* infections, have been comparatively little investigated in infections *in vivo*, compared with CD8^+^ T cell responses to the liver stages of malaria [38], despite the fact that it is the blood stages and CD4^+^ cells which cause disease.

Here, we have used a mouse model of malaria, *P. chabaudi*, to investigate whether *Plasmodium*-specific CD4^+^ T cell memory develops in a blood-stage infection. We show that CD4^+^ T cells with phenotypic characteristics of memory cells are indeed generated within two months of an infection. They develop slowly, and the predominant memory cells are early effector memory cells, the majority of which are able to produce cytokines on short-term re-stimulation and to become effector cells on re-infection. Importantly, we showed that chronically stimulated memory T cells protect immunocompromised mice from infection better than rested memory cells.

There was a striking predominance of CD4^+^CD62L^lo^ effector memory cells remaining after the acute *P. chabaudi* infection, which remained in a relatively activated state. Such cells have also been shown to predominate in the liver stages of malaria [15], [39] and are present in humans protected against malaria by *P. falciparum* sporozoite challenge [40] as well as in other chronic infections such as LCMV [26], *T. cruzi* [13], [14] *T. muris* [41], gamma herpes virus [42], and human Hepatitis B and C [43], and in some cases are critically important in effector sites such as the lung [44], [45], [46].

The type of memory cells maintained may depend on persistence of specific or non-specific stimulation. Studies of CD8+ T cell memory in human viral infections have correlated memory phenotypes with the level of chronic infection [19], and suggested that memory T cell differentiation progresses through a maturation process dependant on the level and duration of antigenic stimulation [17], [18], [47]. Thus a linear pathway of differentiation (Tcm>TemE>TemL) has been proposed based on surface markers and the length of the telomeres in each subset; where low level chronic infections would lead to maintenance of early effector memory cells, but high levels would enhance late effector memory [18]. Here we show conclusively that in chronic infection central memory CD4+ T cells can produce these other two subsets that are indeed related in a linear manner to one another as suggested by the human studies as well as effector T cells. It is likely that selection for high affinity clones and regulation of cytokine production [48] occurs during this differentiation process.

Both Teff and Tem CD4 T cells can protect in various infectious disease models [12], [16], [28], [41], [49], and for CD8 T cells, in some cases, they are more potent than resting memory cells [41], [50], [51]. Although effector memory cells are likely to be more short-lived than central memory cells [52], there are other reports of long-term persistence of effector-type cells [53]. From our studies it appears that CD4^+^ Tem survive the acute infection, especially CD27^+^ cells. This molecule, a member of TNFR family, has been shown to prolong the life of T cells [54]. Extrapolating these findings to human *Plasmodium* infections, it is possible that TemE may survive and protect for extended periods after an infection.

We compared memory CD4 T cells generated and maintained in chronic infection, with those that survived after drug-clearance of infection. We identified an increase in CD44^hi^IL-7Rα^+^ memory CD4 T cells after drug treatment. Although IL-7 can be utilized by memory T cells for survival [55], the observation that in chronically infected mice there were more IL-7Rα^−^ effector cells two months after infection suggests that a sub-population in infected mice may depend instead on antigen or infection for maintenance.

In an adoptive transfer and infection system, chronically stimulated memory CD4^+^ T cells were more protective; they slowed parasite growth, reduced peak parasitemia and were associated with less pathology compared with resting memory cells obtained from infected and drug-cured mice. Despite the eventual higher antibody titers in mice receiving chronically stimulated CD4^+^ T cells, it is unlikely that antibody was the mechanism of parasite control, as at this early stage of infection anti-malaria antibodies were not detectable. However, the chronically stimulated CD4^+^ T cells contained more effector cells and a greater proportion of TNFα^+^IFN-γ^+^IL2^−^ double-producing cells than resting memory cells. Furthermore, mice receiving the chronically stimulated CD4^+^ T cells had significantly greater plasma levels of IFN-γ, and TNFα, cytokines known to play a role in early parasite control [56]. Together, our data suggest that Th1 memory cells can control the acute *P. chabaudi* parasitemia. It will be important to determine which of the activated memory cell subsets [17], [28] within the chronically activated T cell population containing the IFN-γ^+^TNFα^+^IL2^−^ cells are responsible for these anti-parasite effects. The lesser pathology observed in mice receiving chronically stimulated memory T cells compared with rested memory cells, may be the result of the lower parasitemia brought about through the effects of TNFα and IFN-γ, and to the increased levels of IL-10, a cytokine known to down-regulate pathology associated with *P. chabaudi* infections [35].

Here we have seen that the changes in a memory population on withdrawal of infectious stimuli for a month after infection, have subtle effects on the proportions of individual memory T cell subsets in the spleen in *P. chabaudi* malaria, but nevertheless reduce the potential for Th1 cytokine production, and protection. Our data suggest that chronically stimulated memory CD4^+^ T cells are the most protective for controlling early parasitemia and pathology, as seen in *Leishmania* infections, another low-level chronic infection. However Tem may be short-lived, as they are not thought to be the main component of long-lived memory [57]. This might explain why protective memory is lost in mice over time [5] and in humans who move away from endemic areas [3], as previously activated cells revert to a resting state [11]. A contribution of continually stimulated effector memory CD4^+^ T cells to parasite control and regulation of pathology are very much in line with observations that the best protection of humans in areas of endemic malaria is a certain level of continuous exposure [2], [37], and with the association of effector memory cells producing multiple cytokines observed in humans experimentally infected with *P. falciparum* sporozoites [40]. This suggests that vaccination methods that enhance production, and survival of cells which maintain effector functions, may be the most successful in protection against severe malaria, although this must be balanced with the well-tuned production of regulatory cytokines.

## Methods

### Mice and parasites

Female BALB/c (MRC strain) were maintained in the breeding facilities of the MRC National Institute of Medical Research. BALB/c *rag2*−/− mice (RAG°) were a gift from Dr Anton Rolink (University of Basel, Basel, Switzerland). Thy1.1 BALB/c congenic mice (N = 15 BALB/c) were a kind gift of Dr. David Tough, (Jenner Institute, Compton, UK) and were further backcrossed four generations to BALB/c (MRC) for adoptive transfers. B5 TCR Transgenic mice were generated as described [25]. The B5 TCR recognizes MSP1 (1157–1171, ISVLKSRLLKRKKYI/I-E^d^); B5 TCR Tg mice were typed using correct primers Va2, gaacgttccagattccatgg and atggacaagatcctgacagcatcg; and Vβ8.1, cagagaccctcaggcggctgctcagg and atgggctccaggctgttctttgtggttttgattc.

Mice, 5–8 weeks, were infected with 10^5^ *P. chabaudi chabaudi* (AS)–infected erythrocytes i.p. and monitored by Giemsa-stained blood films [22]. The presence of sub-patent chronic parasitemias were determined by subinoculation of blood into naïve recipient mice at various times during the chronic infection as described [6]. To eliminate chronic infections, mice were treated three times with 50mg/kg Chloroquine (Sigma, Dorset, UK) in saline (Sigma) alternate days between days 30–34. *P. chabaudi* (AS) is sensitive to chloroquine at low parasite density, as on day 30 post-infection [6]. Weight (g) was measured on a balance and percent change was calculated as: ((weight - d0 wt)/d0 wt)×100%. Temperature change was recorded from subcutaneously implanted transponders on BioMedic Data Systems DAS5002 (PLEXX, Elst, Netherlands).

### Ethical statement

All animal experiments were carried out according to UK National guidelines (Scientific Procedures) Act 1986 under licence PPL80/2385 approved by the British Home Office. The procedures used were approved by the NIMR institutional Ethical Review Panel.

### Flow cytometry

Single-cell suspensions of spleen, liver and lymph nodes were made in HBSS, incubated in red blood cell lysis buffer (Sigma), and stained in PBS 2% FBS (PAA Laboratories, Somerset, UK) and 0.1% sodium azide with anti-CD16/32 (24G2) supernatant followed by combinations of FITC–, PE–, PE-TexasRed, peridinin chlorophyll protein (PerCP), PE/Cyanine 7 (Cy7), Pacific Blue (PB), biotin- (-b-), allophycocyanin (APC)–, or APC/Cy7-conjugated antibodies: CD4 (RM4-5, BD Biosciences, Cambridge Biosciences Oxford, UK) or CD127-PE, CD90.2-eFluor450, -PE/C7 (A47E, 53-2.1 eBiosciences, Hatfield, UK) or CD43-PE/Cy7 (1B11 Biolegend/Cambridge Bioscience, Cambridge, UK) or CD4-Pacific Orange (PO, Caltag, Invitrogen Paisley, Scotland). The second-step reagents streptavidin-PerCP; (BDbiosciences) and strepdavidin-PB (Invitrogen) were used. For intracellular cytokine staining, cells were pre-treated for one hour with 80–100uM TAPI-2 (Peptides International) [33], and then stimulated for 5h in complete Iscove's medium (cIMDM, Sigma), 10% FBS, 2 mM L-glutamine, 0.5 mM sodium pyruvate, 100 U penicillin, 100 µg streptomycin, and 50 µM 2-ME (Gibco, Invitrogen) with PMA (50 ng/mL; Sigma), ionomycin (500 ng/mL; Sigma), and BrefeldinA (10 µg/mL; Sigma) for the last two hours. Cells were fixed in 2% paraformaldehyde 20 minutes, and resuspended in staining buffer overnight. Permeabilized in Perm/Wash buffer (BDbiosciences) 25 minutes and washed twice, then incubated for 40 minutes with anti–IFNγ-PE (XMG1.2), IL2-APC (JES6-5H4), TNFα (MP6-XT22), IL10-APC (JES5-16E3; all from BD Biosciences). Cells were washed thrice in Perm/Wash and resuspended in staining buffer. Cells were collected on a FACSCalibur or 9-color CyAn ADP (DAKO, Beckman Coulter) using Summit software (Cytomation) and analyzed in FlowJo (Tree Star, Ashland, OR). Staining combinations were designed through a meticulous optimization strategy as described by Seder *et al* and Tung *et al* [36], [58]. Compensation was performed in Flow Jo using single stained splenocytes or bead controls (BD CompBeads Anti-Rat Ig, κ) and results thoroughly checked for accuracy and consistency. For presentation, data from 2–5 mice is concatenated to achieve sufficient cell numbers (5–10,000) for presentation and Boolean gating analysis, only after each mouse was carefully analyzed and averages and standard errors of the mean (SEM) calculated.

For *in vitro* stimulation, naïve cell transfers, and RAG° transfers, B5 Tg CD4^+^ cells were purified by positive selection with magnetic micro-beads (Miltenyi Biotec, Surrey, UK) using magnetic-activated cell sorting (autoMACS) and further purified as naïve (CD44^lo^, CD25^−^) or memory (CD44^hi^) T cells by high speed (MoFlo, Cytomation, Beckman Coulter) sorting to >99% purity. In naïve cell transfers, cells were labeled for 10 min at 37°C with 1µM CFSE (Molecular Probes, Invitrogen) after washing thrice in cation-free PBS. Memory subsets were purified on a FACS Aria (BDbiosciences) as shown in **Figure S4**, as CD44^hi^ IL-7Rα^+^, and Tcm, CD62L^hi^ CD27^+^; TemE, CD62L^lo^ CD27^+^; and TemL, CD62L^lo^ CD27^−^ to about 95% purity.

### Adoptive transfers

Splenic CD4^+^ T and CD19^+^ B cells were purified by MACS (95% purity, Miltenyi). B5 Tg CD4^+^ T cells were transferred (2×10^6^) into uninfected congenic Thy1.1 BALB/c mice. To determine presence of B5 antigen, CFSE-labeled CD4^+^ B5 T cells were transferred into previously infected mice for 4–5 days. Immune B cells were generated by infecting BALB/c mice with 10^5^ *P. chabaudi* twice, 2 months apart. In RAG° transfer, 10^5^ naïve, memory, or memory subsets T cells and 10^7^ B cells were sort-purified (MoFlo, cytomation) and transferred. Reconstitution was analyzed day 39 post infection and all mice had 3.5–28% (of lymphocyte gate) T cells (naïve T cells average 19.5±2.4%, Tmem average 11±1.0%) and 0.8–5.3% B cells in the spleen (average 2.3±0.2%). One mouse with less than 0.5% of both B and T cells, and excessive splenomegaly was excluded from the study.

### Serum cytokines

IL-10, TNF and IFN-γ were measured in serum from animals at day 7 post-infection using the Luminex Flexible Beadlyte Immunoassay (BioRad or Upstate/Millipore, Milton Keynes, UK), according to the manufacturers instructions. Plates were read and analyzed using the Luminex 100 machine and software (Upstate).

### Malaria-specific ELISA

Malaria-specific antibodies were measured by ELISA, as described. Plates were coated with a soluble fraction of the lysate of *P. chabaudi* blood-stage parasites, IgG was detected using alkaline-phosphatase linked secondary antibody (Southern Biotech, Cambridge, UK) and PNPP substrate (Sigma). Results are expressed as relative units based on a standard hyperimmune plasma (all described in [59]).

### Statistics

Statistics were performed in Prism (GraphPad, La Jolla, CA) using Student *t*-test, with *P*≤.05 was considered significant.

## Supporting Information

Figure S1 CD44 gating is shown clearly as correlating with division, as shown in **A)** which is gated on Thy1.2^+^CD4^+^ cells. **B)** CD4^+^ cells show a double peak for CD44 staining (red), while CFSE^+^ B5 Tg cells are uniformly CD44^hi^ (blue) and CFSE^neg^ B5 Tg cells are CD44^lo^ (green). The CD44 gate was set using this type of analysis for each sample.
(0.19 MB TIF)

Figure S2 As few as 5,000 CD4+ naive-purified CFSE-labeled B5 TCR Tg T cells were transferred into Thy1.1 congenic mice, which were infected with 10^5^ *P. chabaudi*. B5 T cells were identified after MACS enrichment of Thy1.2^+^ cells by using a high-sensitivity double labeling approach. The technique is specific as evidenced by the CFSE labeling of the identified Thy1.2^+^ B5 cells on day 6. **A)** On day 6 post-infection (pi) with transfers of 5×10^4^, Teff were generated in the spleen, but not in the lymph nodes. **B)** On day 60 pi as few as 5,000 cells were still detectable by this method and seem to have a similar phenotype as when 2×10^6^ are transferred, as in the main figures. B5 Tg cells collected from two mice were concatenated to make these plots. More cells divided when fewer were transferred, as previously reported. Cells were not detectable, even using this high-sensitivity double fluorochrome labeling, without infection (1×10^6^ uninf), at day 60 pi.
(0.46 MB TIF)

Figure S3 Effector CD4+ T cells persist in *P. chabaudi* infection and early effector memory cells predominate. Naïve (CD44^lo^CD25^−^), CFSE-labeled B5 T cells (2×10^6^) were seeded into congenic Thy1.1 mice, which were then infected with 10^5^ *P. chabaudi* iRBC. **(Top)** CD44^hi^IL-7Rα(CD127)^+^ memory cells are shown in the divided CFSE^neg^ population. **(Bottom)** Tmem were subdivided using CD62L and CD27 to measure central (Tcm, CD62L^hi^CD27^+^), and early effector memory cells (TemE, CD62L^lo^CD27^+^) as well as CD27^−^ late effector memory T cells (TemL, CD62L^lo^CD27^−^) shown here at day 60 of infection. These are the plots that were concatenated to show CD4^+^Thy1.2^+^CFSE^neg^ cells on day 60 post-infection in Figure 2. Gated as shown in Figure 1A). Mouse 2 was not included in further analysis due to poor recovery. Experiment was repeated three times with similar results.
(0.71 MB TIF)

Figure S4 By d45 Tem predominate in liver and are also present in lymph nodes A) Naive sorted CD4^+^CD44^lo^CD25^−^ B5 T cells were transferred (2×10^6^) into congenic Thy1.1 recipients, which were then infected. Divided B5 cells (CD4^+^Thy1.2^+^CFSE^neg^) are analyzed for CD44, CD62L and IL-7Rα to observe effector cell kinetics with percentages of Tmem (CD44^hi^ IL-7Rα^+^), Teff (CD62L^lo^ IL-7Rα^−^), Tcm (CD44^hi^CD62L^hi^), Tem (CD44^hi^CD62L^lo^, includes some Teff) in the **A)** Liver or **B)** peripheral lymph nodes (pooled). Data represents concatenated total B5 cells from 2–5 mice (>1000 cells/timepoint).
(0.12 MB TIF)

Figure S5 Non-transgenic and B5 transgenic effector CD4^+^ T cells persist in *P. chabaudi* infection. BALB/c or B5 TCR Tg mice were infected with 10^5^ *P. chabaudi* iRBC and were analyzed for the presence of effector and memory T cells throughout infection. **A)** IL-7Rα^−^CD62L^lo^ effector cells and **B)** CD44^hi^IL-7Rα^+^ memory cells are shown in the CD4^+^ population. The kinetics of effector cell and memory cell generation is remarkably similar in these two strains of mice validating the use of MSP-1 specific B5 Tg memory cells for differentiation experiments.
(0.68 MB TIF)

Figure S6 B5 TCR Tg CD4+ cells were MACS purified to >90% purity, then purified on a BDFACSAria as CD44^hi^ IL-7Rα+ memory cells and Tcm (CD27^+^CD62L^hi^), TemEarly (CD27^+^CD62L^lo^) and TemLate (CD27^−^CD62L^lo^) for RAG transfer in Figure 4.
(0.64 MB TIF)

Figure S7 Chloroquine treatment of chronic infection reduces available T cell antigen and protection. **A)** BALB/c mice were infected with 10^5^ *P. chabaudi*-iRBC and treated days 30–34 with Chloroquine (CQ) to clear the persistent infection. Some mice were re-infected at day 45 (10^5^ parasites). Parasitemia was measured as % parasitized RBC/total RBC. B) To detect antigen availability in previously infected mice CFSE labeled B5 Tg CD4^+^ T cells were transferred for 4 days into mice infected 45 or 60 days previously. Data are presented as the average of divided cells (%CFSE^neg^) from infected mice with background division in paired, uninfected mice subtracted. Error bars represent SEM of 5 mice per group.
(0.11 MB TIF)

Figure S8 Effector CD4+ T cells persist in chronic *P. chabaudi* infection. Naïve (CD44^lo^CD25-), CFSE-labeled B5 T cells (2×10^6^) were seeded into congenic Thy1.1^+^ mice, which were then infected with 10^5^ *P. chabaudi* iRBC. CD44^hi^ IL-7Rα^+^ memory cells are shown and effector cells (IL-7Rα-) are shown here at day 60 of infection in both the MSP-1 specific B5 transgenic cells and the endogenous CD4+ T cells. −CQ indicates no chloroquine treatment, +CQ indicates treatment with chloroquine at day 30 to remove the chronic infection.
(0.15 MB TIF)
