# Supplementary figures and images for: Effector Memory Th1 CD4 T Cells Are Maintained in a Mouse Model of Chronic Malaria
Source: PLoS Pathog. 2010 Nov 24;6(11):e1001208. doi: 10.1371/journal.ppat.1001208 (PMC2991260; doi:10.1371/journal.ppat.1001208)

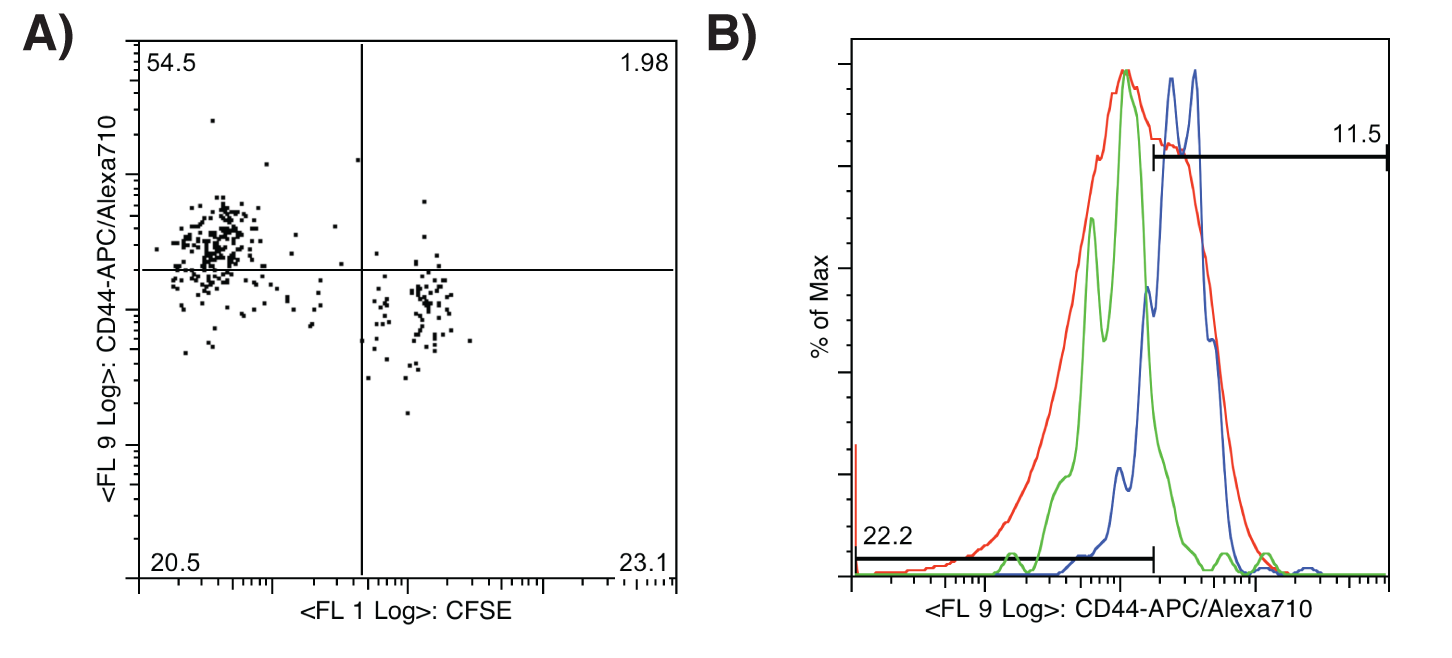

Supplement: Figure S1 — CD44 gating is shown clearly as correlating with division, as shown in A) which is gated on Thy1.2+CD4+ cells. B) CD4+ cells show a double peak for CD44 staining (red), while CFSE+ B5 Tg cells are uniformly CD44hi (blue) and CFSEneg B5 Tg cells are CD44lo (green). The CD44 gate was set using this type of analysis for each sample. (0.19 MB TIF) [file ppat.1001208.s001.tif]

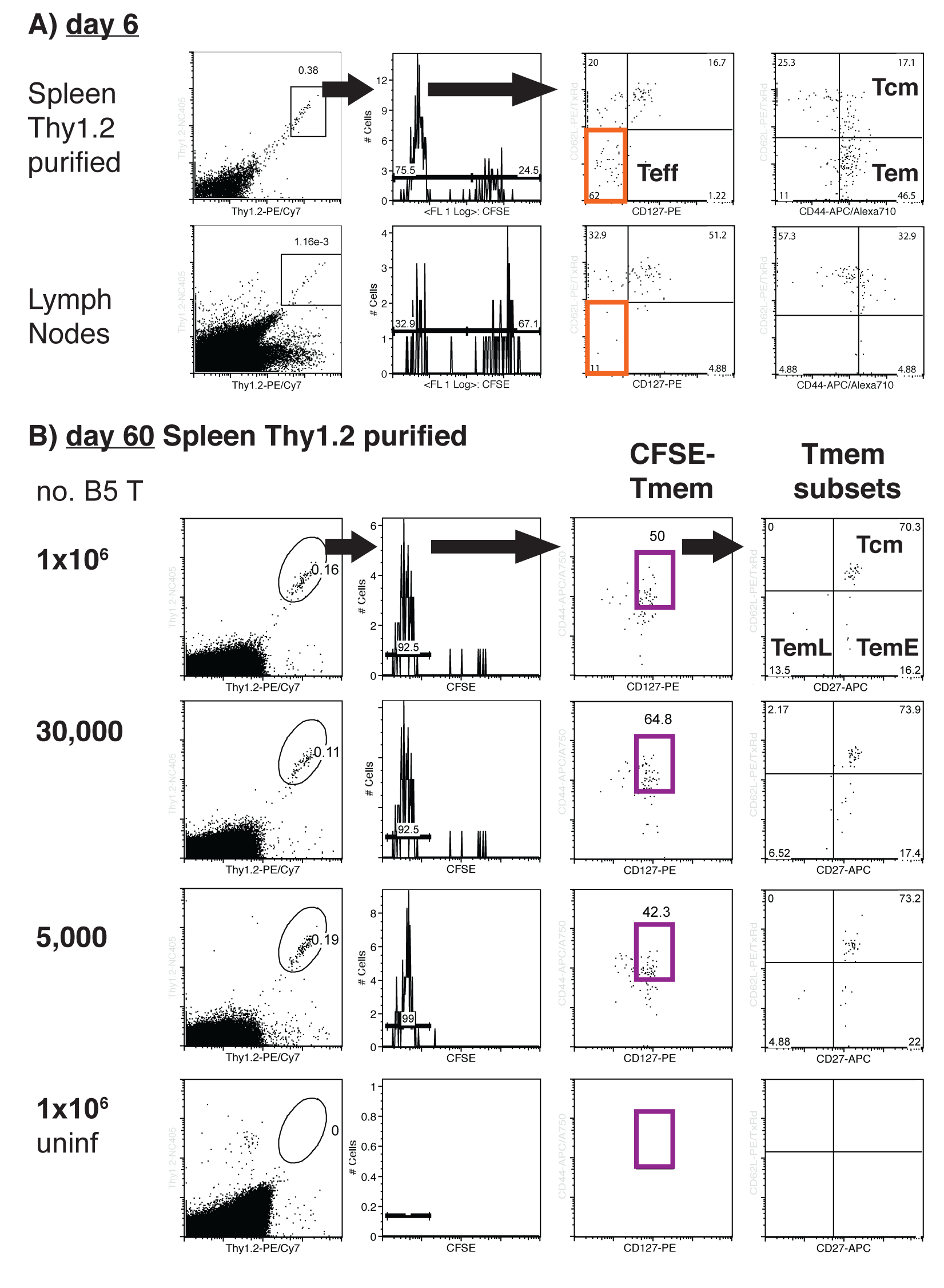

Supplement: Figure S2 — As few as 5,000 CD4+ naive-purified CFSE-labeled B5 TCR Tg T cells were transferred into Thy1.1 congenic mice, which were infected with 105 P. chabaudi. B5 T cells were identified after MACS enrichment of Thy1.2+ cells by using a high-sensitivity double labeling approach. The technique is specific as evidenced by the CFSE labeling of the identified Thy1.2+ B5 cells on day 6. A) On day 6 post-infection (pi) with transfers of 5×10^4^, Teff were generated in the spleen, but not in the lymph nodes. B) On day 60 pi as few as 5,000 cells were still detectable by this method and seem to have a similar phenotype as when 2×10^6^ are transferred, as in the main figures. B5 Tg cells collected from two mice were concatenated to make these plots. More cells divided when fewer were transferred, as previously reported. Cells were not detectable, even using this high-sensitivity double fluorochrome labeling, without infection (1×10^6^ uninf), at day 60 pi. (0.46 MB TIF) [file ppat.1001208.s002.tif]

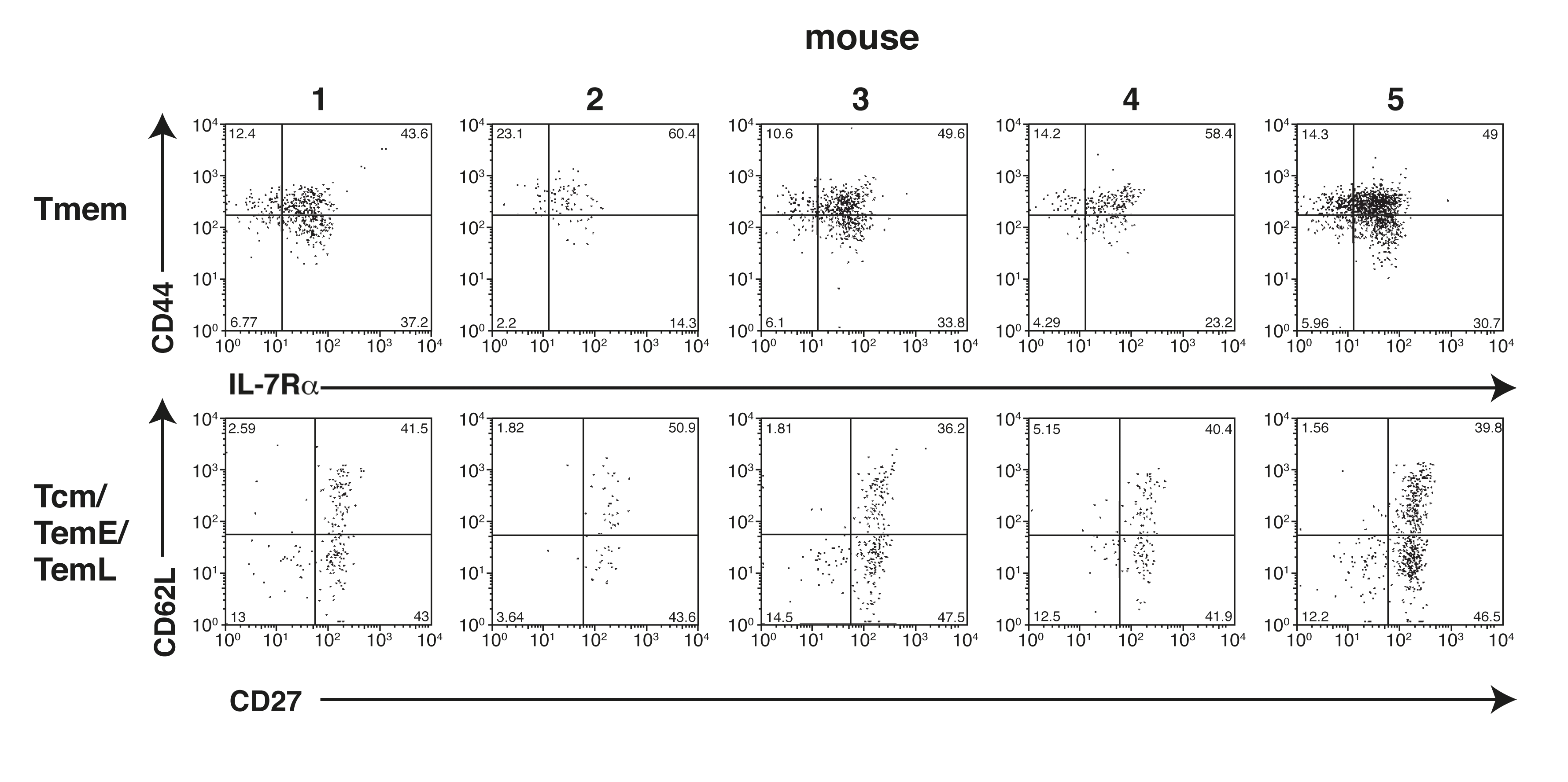

Supplement: Figure S3 — Effector CD4+ T cells persist in P. chabaudi infection and early effector memory cells predominate. Naïve (CD44loCD25−), CFSE-labeled B5 T cells (2×10^6^) were seeded into congenic Thy1.1 mice, which were then infected with 105 P. chabaudi iRBC. (Top) CD44hiIL-7Rα(CD127)+ memory cells are shown in the divided CFSEneg population. (Bottom) Tmem were subdivided using CD62L and CD27 to measure central (Tcm, CD62LhiCD27+), and early effector memory cells (TemE, CD62LloCD27+) as well as CD27− late effector memory T cells (TemL, CD62LloCD27−) shown here at day 60 of infection. These are the plots that were concatenated to show CD4+Thy1.2+CFSEneg cells on day 60 post-infection in Figure 2. Gated as shown in Figure 1A). Mouse 2 was not included in further analysis due to poor recovery. Experiment was repeated three times with similar results. (0.71 MB TIF) [file ppat.1001208.s003.tif]

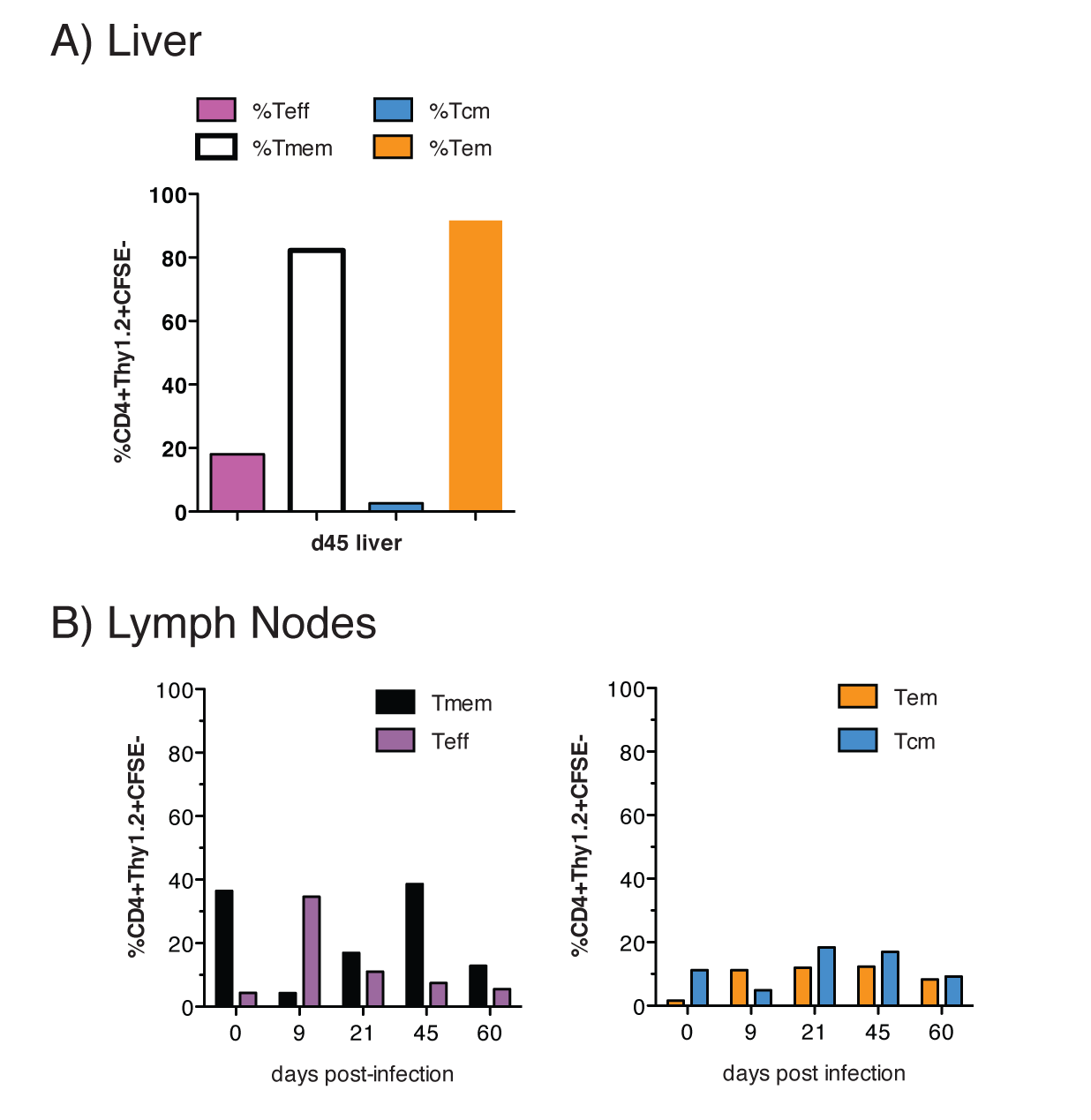

Supplement: Figure S4 — By d45 Tem predominate in liver and are also present in lymph nodes A) Naive sorted CD4+CD44loCD25− B5 T cells were transferred (2×10^6^) into congenic Thy1.1 recipients, which were then infected. Divided B5 cells (CD4+Thy1.2+CFSEneg) are analyzed for CD44, CD62L and IL-7Rα to observe effector cell kinetics with percentages of Tmem (CD44hi IL-7Rα+), Teff (CD62Llo IL-7Rα−), Tcm (CD44hiCD62Lhi), Tem (CD44hiCD62Llo, includes some Teff) in the A) Liver or B) peripheral lymph nodes (pooled). Data represents concatenated total B5 cells from 2–5 mice (>1000 cells/timepoint). (0.12 MB TIF) [file ppat.1001208.s004.tif]

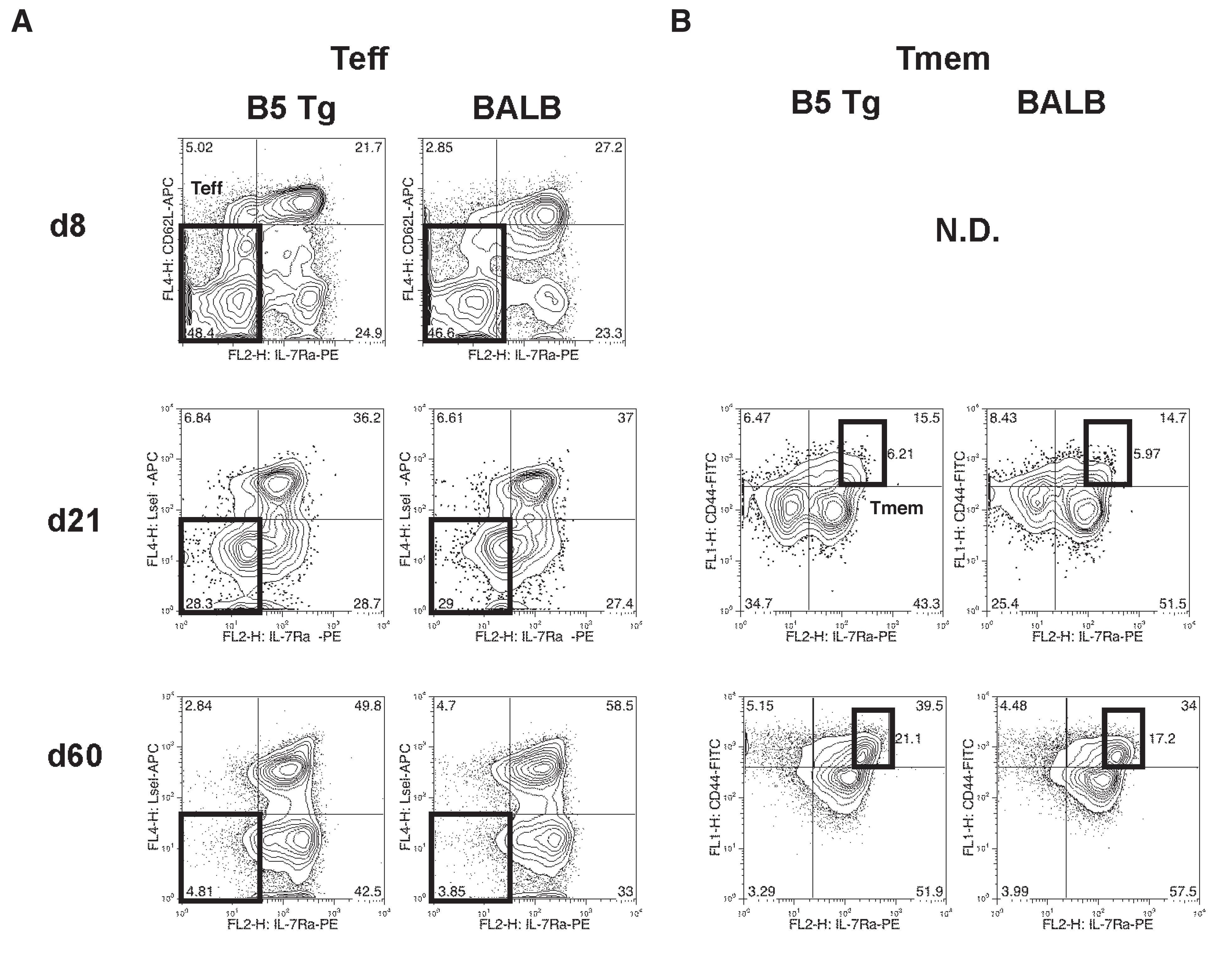

Supplement: Figure S5 — Non-transgenic and B5 transgenic effector CD4+ T cells persist in P. chabaudi infection. BALB/c or B5 TCR Tg mice were infected with 105 P. chabaudi iRBC and were analyzed for the presence of effector and memory T cells throughout infection. A) IL-7Rα−CD62Llo effector cells and B) CD44hiIL-7Rα+ memory cells are shown in the CD4+ population. The kinetics of effector cell and memory cell generation is remarkably similar in these two strains of mice validating the use of MSP-1 specific B5 Tg memory cells for differentiation experiments. (0.68 MB TIF) [file ppat.1001208.s005.tif]

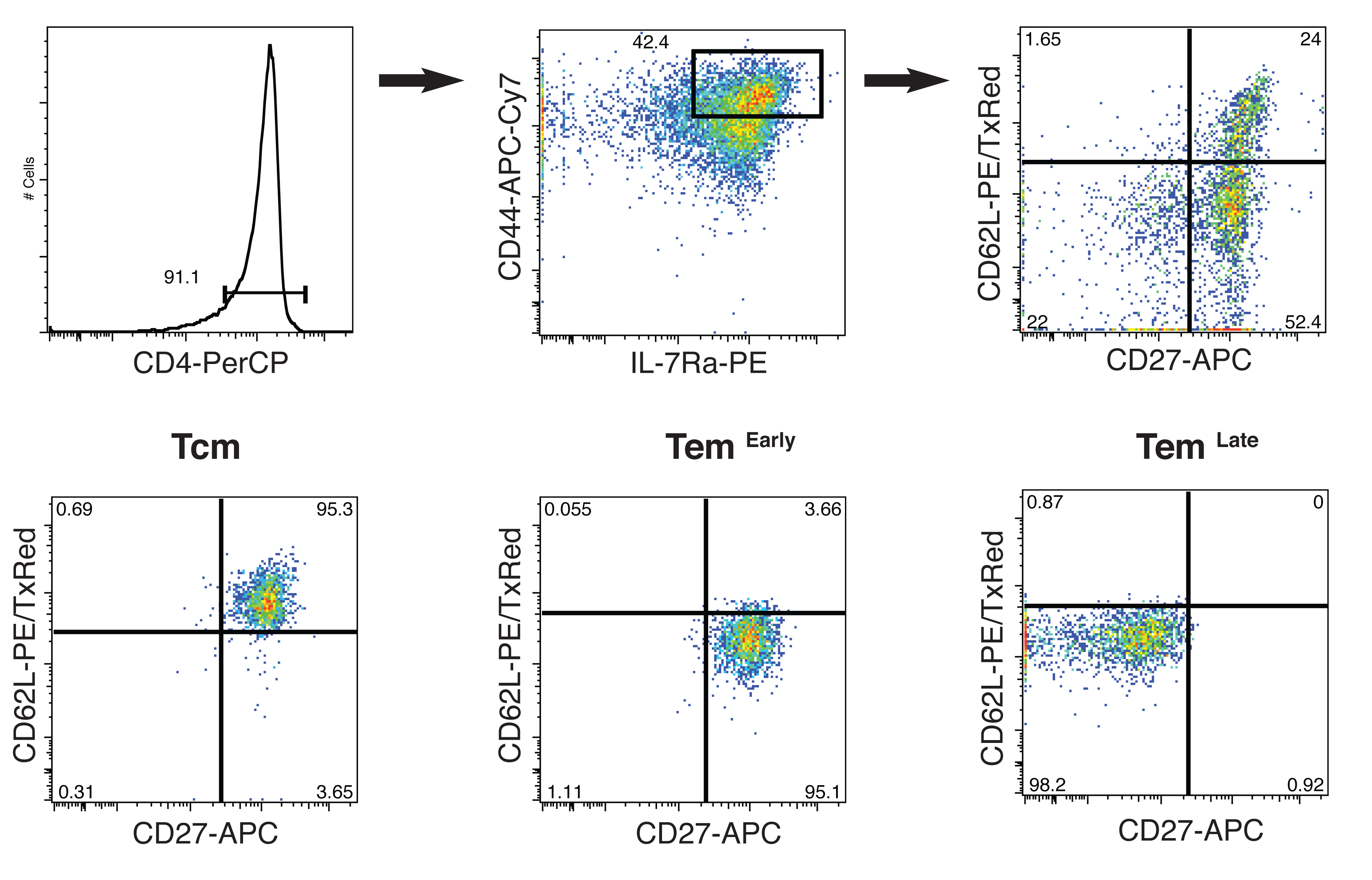

Supplement: Figure S6 — B5 TCR Tg CD4+ cells were MACS purified to >90% purity, then purified on a BDFACSAria as CD44hi IL-7Rα+ memory cells and Tcm (CD27+CD62Lhi), TemEarly (CD27+CD62Llo) and TemLate (CD27−CD62Llo) for RAG transfer in Figure 4. (0.64 MB TIF) [file ppat.1001208.s006.tif]

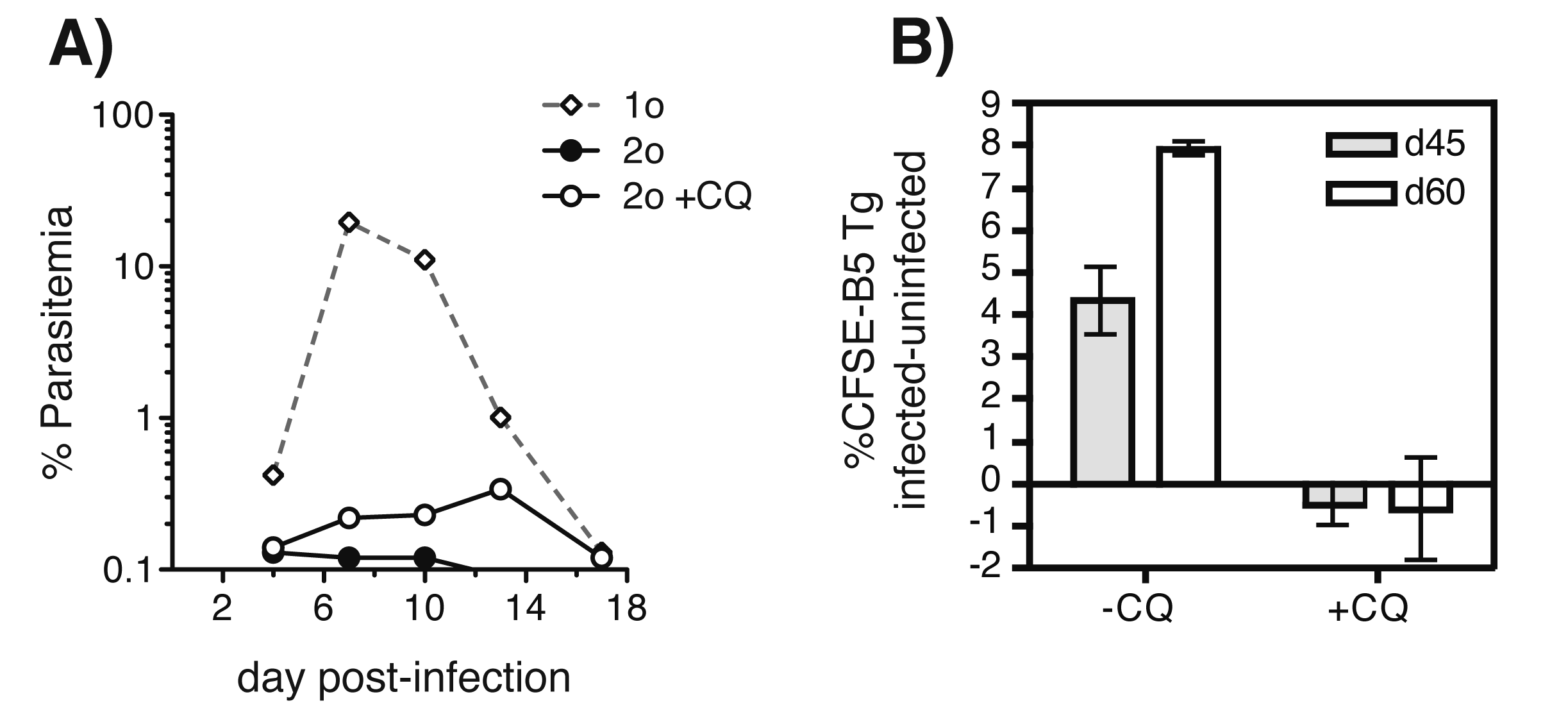

Supplement: Figure S7 — Chloroquine treatment of chronic infection reduces available T cell antigen and protection. A) BALB/c mice were infected with 105 P. chabaudi-iRBC and treated days 30–34 with Chloroquine (CQ) to clear the persistent infection. Some mice were re-infected at day 45 (105 parasites). Parasitemia was measured as % parasitized RBC/total RBC. B) To detect antigen availability in previously infected mice CFSE labeled B5 Tg CD4+ T cells were transferred for 4 days into mice infected 45 or 60 days previously. Data are presented as the average of divided cells (%CFSEneg) from infected mice with background division in paired, uninfected mice subtracted. Error bars represent SEM of 5 mice per group. (0.11 MB TIF) [file ppat.1001208.s007.tif]

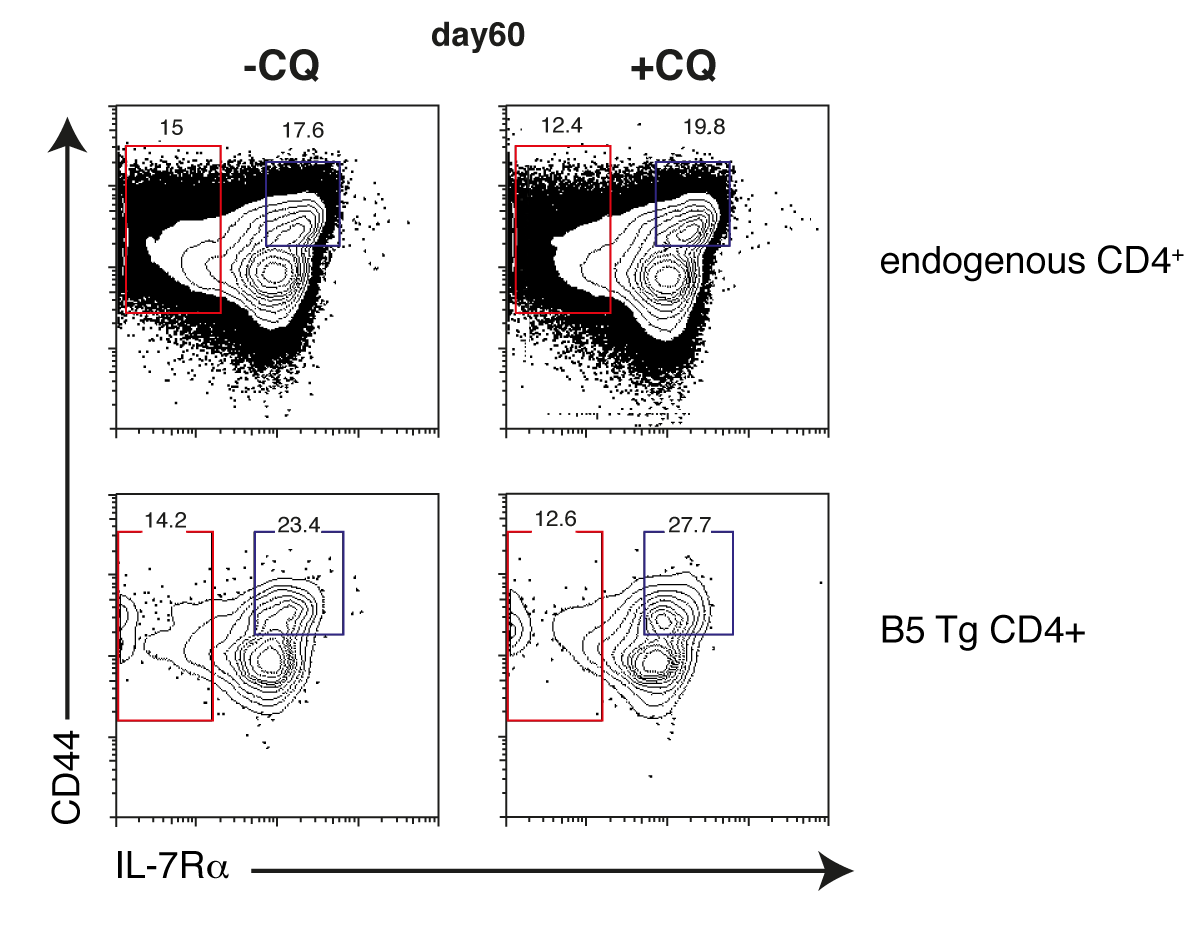

Supplement: Figure S8 — Effector CD4+ T cells persist in chronic P. chabaudi infection. Naïve (CD44loCD25-), CFSE-labeled B5 T cells (2×10^6^) were seeded into congenic Thy1.1+ mice, which were then infected with 105 P. chabaudi iRBC. CD44hi IL-7Rα+ memory cells are shown and effector cells (IL-7Rα-) are shown here at day 60 of infection in both the MSP-1 specific B5 transgenic cells and the endogenous CD4+ T cells. −CQ indicates no chloroquine treatment, +CQ indicates treatment with chloroquine at day 30 to remove the chronic infection. (0.15 MB TIF) [file ppat.1001208.s008.tif]
